# Patient-Centric Approaches for Phase I Combination Trials Come on Stage

**DOI:** 10.1158/2159-8290.CD-23-0534

**Published:** 2023-06-03

**Authors:** Alberto Hernando-Calvo, Elena Garralda

**Affiliations:** 1 Vall d'Hebron University Hospital, Barcelona, Spain.; 2 Vall d'Hebron Institute of Oncology, Barcelona, Spain.

## Abstract

A disruptive clinical trial design allowed Drilon and colleagues to demonstrate proof of concept of the potential of PF-07284892 to overcome resistance mechanisms to targeted therapies in the clinic.

*See related article by Drilon et al., p. 1789 (7).*

The heterogeneity of cancer molecular profiles and the widespread variability of anticancer responses has prompted the need to tailor therapies to individual genomic profiles. Although different molecularly guided therapeutics targeting oncogene addiction have become the standard-of-care treatment of different cancers, single-agent activity is hampered by the development of resistance and patients receiving targeted agents will inevitably end up progressing. In this setting, novel rational combinations are poised to become key in overcoming intrinsic and acquired resistance mechanisms while prolonging the duration of clinical benefit achieved for these agents when used as monotherapies.

The June 2012 FDA approval of the combination of pertuzumab and trastuzumab with docetaxel for HER2-positive breast cancer marked the first approval of a combination therapy involving multiple targeted therapies. More than a decade has passed, and the number of preclinical publications exploring different combinations of targeted therapies is rapidly expanding. However, only very few have reached routine clinical care. As described in Fig. 1, multiple reasons underlie the scarcity of targeted therapy combinations into the clinic. Challenges associated with the development of novel treatment combinations include biomarker discovery for patient selection, development of clear scientifically rational combinations, the potential for additive toxicities, and the constraints of established clinical trial development paradigms and regulatory requirements (1).

**Figure 1.**
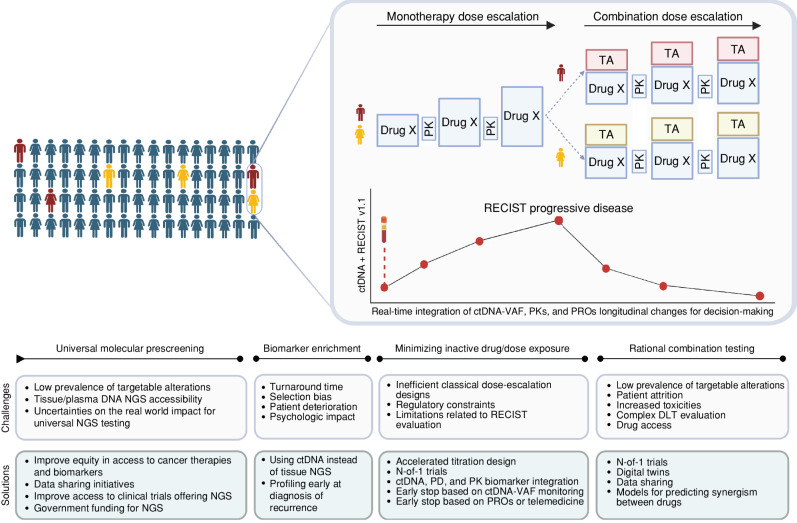
Challenges and potential solutions for the implementation of the next generation of phase I clinical trials for precision oncology adapted from Drilon and colleagues’ proposed design (7). Universal screening of tumor genomic alterations for patients with advanced solid tumors will be required to maximize the chances of detecting targetable alterations across the population. The implementation of biomarker enrichment strategies will be required to improve efficacy outcomes across early-phase clinical trials. For this purpose, early molecular profiling at recurrence and novel next-generation sequencing (NGS) platforms with shorter turnaround times will be required to avoid patient deterioration. For biomarker-selected populations, clinical trial designs customized to minimize exposure to inactive drugs or subtherapeutic treatment doses could rely on real-time integration of circulating tumor DNA-variant allele frequency (ctDNA-VAF), patient-reported outcomes (PRO), and pharmacokinetic/pharmacodynamic (PK/PD) data in addition to RECIST evaluation. Finally, clinical trials including sequential dose-escalation parts for monotherapies and combinations or N-of-1 trials with the implementation of digital twins and data sharing initiatives could help to optimize rational combination testing (8, 10). DLT, dose-limiting toxicity; TA, targeted agent. Created with BioRender.com.

Over the past few years, and more importantly since the beginning of the COVID-19 pandemic, the drug development field has evolved toward more patient-centered approaches. Examples of this include, but are not limited to, the implementation of virtual appointments as part of phase I clinical trial protocols; definition of patient-reported outcomes (PRO) as objectives across early-phase clinical trials; involving patient advocates early in trial design; or the implementation of the FDA Project Optimus, which is focused on dose optimization for oncology drugs (2, 3). Moreover, precision oncology has already disrupted clinical trial design in outstanding ways, with the appearance of master protocols, including basket and umbrella studies, among other designs. It is becoming clear that developing combinations in an efficient and feasible way will also require the implementation of changes in the way we perform drug development. As an example, traditionally before beginning a combination phase I trial, both agents will have been explored in specific monotherapy trials or cohorts and both should have demonstrated activity as single agent. However, this is particularly challenging in drugs with limited expected efficacy in monotherapy, such as those designed to overcome bypass signaling pathways involved in resistance to targeted agents.

Src homology region 2 domain-containing phosphatase-2 (SHP2) is a phosphatase that acts by transducing signaling from receptor tyrosine kinase (RTK), including EGFR, MET, and HER2 (4). Preclinical data support dual inhibition of the pathway to suppress intrinsic mechanisms of resistance by decreasing MAPK signaling, resulting in decreased tumor cell proliferation and tumor growth (4). Different SHP2 inhibitors have been specifically designed to overcome resistance to RTK- and MAPK-targeted agents, including TNO155 or RMC-4630. In both cases, recently reported dose-escalation data have shown limited activity in monotherapy, which contrasts with the large number of patients treated (5, 6). Overall, this underscores the necessity of novel patient-centric clinical trial designs to preclude and minimize the exposure of patients to subtherapeutic doses or agents with limited monotherapy activity.

In this issue of *Cancer Discovery*, Drilon and colleagues provide preclinical data and preliminary results from a phase I clinical trial evaluating a new allosteric SHP2 inhibitor (PF-07284892; ref. 7). PF-07284892 was tested as a single agent and in combination for patients with advanced solid tumors that have developed resistance to an oncogene-matched targeted therapy (NCT04800822). This was a phase I dose-escalation clinical trial with a novel and disruptive design encompassing two parts. In the first part, PF-07284892 was escalated following a Bayesian dose-escalation methodology. Those patients with progressive disease after PF-07284892 treatment were then allowed to continue on trial in the combination dose-escalation part. Patients then received a combination of PF-07284892 with a specific molecularly guided therapy (lorlatinib, encorafenib + cetuximab or binimetinib) based on their genomic profiles. Eligibility criteria included patients with non–small cell lung cancer (NSCLC) with *ALK* or *ROS1* gene fusions, patients with colorectal cancer with *BRAF*^V600E^ mutations, and any other solid tumor with *RAS, NF1*, or *BRAF* class 3 mutations. Other *ROS1*-positive solid tumors were also considered after discussion with the sponsor. Cycle duration was 21 days, image assessments were performed every 2 cycles and circulating tumor DNA (ctDNA) longitudinal assessments every 2 to 4 cycles. Primary outcome measures were incidence of dose-limiting toxicities for both PF-07284892 single agent or combinations, toxicity data, and, importantly, overall response rate.

Eight patients were treated in dose level 1, with one patient being nonevaluable due to increase in alanine aminotransferase and bilirubin leading to PF-07284892 monotherapy discontinuation. One additional patient did not transition to receive the combination therapy due to rapid progression on PF-07284892 monotherapy, and one patient developed a cetuximab-induced infusion reaction leading to treatment interruption. Among the five patients who received PF-07284892–based combinations, one patient with NSCLC and *EML4–ALK* fusion and another with pancreatic cancer and *GOPC–ROS1* fusion received treatment with PF-07284892 and lorlatinib, achieving confirmed partial responses, and one patient with *KRAS*^G12D^-mutated ovarian cancer received binimetinib as a combination partner of PF-07284892, also achieving a confirmed partial response. One additional patient achieved an unconfirmed partial response with the combination of encorafenib + cetuximab with PF-07284892. Finally, a patient with *KRAS*^G12D^-mutated colorectal cancer achieved disease stabilization. It is worth highlighting that all patients had previously progressed to the same or similar targeted agent in the past. The authors used an approach based on a Guardant360 73-gene targeted panel for real-time variant allele frequency (VAF) monitoring. High-throughput technologies such as next-generation sequencing and ctDNA enabled longitudinal assessment of tumor burden kinetics and early recognition mechanisms of resistance.

To the best of our knowledge, these results represent the first attempt to optimize a dose-escalation clinical trial design to include a first-in-human dose-defining part for a single-agent targeted therapy followed by treatment with a personalized combination according to a specific genomic profile. In line with this approach, N-of-1 clinical trials, in which single patients act as his or her own control, have been in the spotlight recently as potential patient-centric clinical trial designs. N-of-1 clinical trials using novel molecular profiling platforms offer a unique opportunity to test treatment combinations in the setting of low-prevalence predictive biomarkers or rare cancers (8). However, regulatory concerns still arise, as fewer data on safety and pharmacokinetic analyses are expected to be gathered with this clinical trial design and questions remain on how to pool data from different single-patient trials to inform drug approval. The model proposed by Drilon and colleagues builds on this approach by providing a customized phase I trial design in which data are already aggregated under one protocol (7). Specifically, this study provides proof of concept of the potential of SHP2 inhibitors to overcome resistance in the clinic to different targeted agents. In addition, it defines an optimized framework for accelerated testing of novel treatment combinations in phase I dose-escalation clinical trials.

Further studies will need to be performed to fully understand the extent of this synergism between PF-07284892 and different targeted therapies, elucidating the best moment to initiate the combination, the role of the response to previous targeted agents, the selected combination partner, the optimal dosage of both drugs to avoid toxicities, and a better definition of the subset of patients in whom durable clinical benefit will be observed. Another important aspect to highlight is the role of ctDNA tracking in this study to monitor the appearance of resistance and disease progression. Undoubtedly, our ability to perform molecular profiling in a timely manner will provide unique opportunities for the use of molecularly guided therapies in selected patient populations and inform unprecedented ways to overcome resistance mechanisms during phase I clinical trials. ctDNA assessed at baseline can have prognostic implications, and on-treatment analyses may help unveil potential targets for precision interception (9). However, multiple challenges and hurdles still need to be addressed to fully understand its role in response assessment. As an example, patient number 7, described in the article as having *KRAS*^G12D^-mutant ovarian cancer, was considered resistant to PF-07284892 due to clinical deterioration despite RECIST v1.1 stability criteria and the fact that *ATM*^L1517P^ VAF appeared to decrease early during monotherapy. Notably, this case highlights how the integration of PROs, ctDNA-VAF, and RECIST assessments will be required for an agile implementation of personalized combinations.

Finally, with the recent advances on data science and machine learning and their advent into the drug development space, the possibilities to test multiple combinations will continue to grow exponentially. While the implementation of digital twins (10), in which a virtual representation of the patient is used to inform clinical investigation, may be a very powerful tool to guide therapeutic choices in the future, the need for novel and more flexible clinical trial designs will still be required to generate evidence that ensures a generalizable and equitable access to novel therapies.

To conclude, combination of novel drugs calls for more individualized clinical trial designs that incorporate real-time tracking of tumor molecular profiles. The data presented by Drilon and colleagues will undoubtedly contribute to the general efforts of implementing patient-centric personalized treatment approaches in the setting of phase I clinical trial designs and methodology.
